# Correction of hypothermic and dilutional coagulopathy with concentrates of fibrinogen and factor XIII: an in vitro study with ROTEM

**DOI:** 10.1186/s13049-014-0073-z

**Published:** 2014-12-16

**Authors:** Dag Winstedt, Owain D Thomas, Fredrik Nilsson, Knut Olanders, Ulf Schött

**Affiliations:** Faculty of Medicine, Lund University, Lund, Sweden; Department of Anaesthesia and Intensive Care, Skåne University Hospital Lund, Lund, 221 85 Sweden; Department of Paediatric Anaesthesia and Intensive Care, Skåne University Hospital Lund, Lund, 221 85 Sweden; Research and Development Centre, Skåne, Skåne University Hospital Lund, Lund, 221 85 Sweden

**Keywords:** Factor XIII, Fibrinogen, Hemodilution, Hypothermia, Hemostasis, Thrombelastography

## Abstract

**Background:**

Fibrinogen concentrate treatment can improve coagulation during massive traumatic bleeding. The aim of this in vitro study was to determine whether fibrinogen concentrate, or a combination of factor XIII and fibrinogen concentrates, could reverse a haemodilution-induced coagulopathy during hypothermia.

**Methods:**

Citrated venous blood from 10 healthy volunteers was diluted in vitro by 33% with 130/0.42 hydroxyethyl starch (HES) or Ringer’s acetate (RAc). The effects of fibrinogen concentrate corresponding to 4 gram per 70 kg, or a combination of the same dose of fibrinogen with factor XIII (20 IU per kg), were measured using rotational thromboelastometry (ROTEM). The blood was analysed at 33°C or 37°C with ROTEM EXTEM and FIBTEM reagents. Clotting time (CT), clot formation time (CFT), alpha angle (AA) and maximal clot formation (MCF) were recorded.

**Results:**

Fibrinogen with or without factor XIII improved all ROTEM parameters in either solution irrespective of temperature, with the exception of EXTEM-AA and EXTEM-CFT in HES haemodilution. Fibrinogen increased FIBTEM-MCF more in the samples diluted with RAc than HES, particularly in presence of factor XIII.

**Conclusions:**

Fibrinogen improved in vitro haemodilution-induced coagulopathy at both 33°C and 37°C, though more efficiently after crystalloid than HES haemodilution. Factor XIII had an additional effect on FIBTEM-MCF, but only after crystalloid dilution.

## Introduction

Haemodilution and hypothermia both contribute to coagulopathy and aggravate acute traumatic coagulopathy, which has been recognized as a significant cause of death in patients with traumatic injuries [1]. We therefore studied the efficacy of concentrates of fibrinogen and factor XIII (FXIII) in improving in vitro coagulopathy induced by haemodilution and hypothermia.

Hypothermia impairs coagulation mainly by platelet inhibition [2], whereas haemodilution principally impairs plasma coagulation [3]. In addition to the dilutional effects seen with crystalloids, synthetic colloid solutions impair fibrinogen polymerization and platelet function [3]. Hypothermia and haemodilution induced coagulopathy has been studied extensively with whole blood viscoelastic haemostatic assays (VHA) such as thromboelastometry (ROTEM), but there are few studies of simultaneous hypothermia and haemodilution [4,5]. In addition, in vitro correction of haemodilution-induced coagulopathy with fibrinogen concentrate has been studied extensively [6,7], but not during hypothermia. Previous in vitro studies indicate an additional effect of high doses of FXIII together with fibrinogen to correct haemodilution-induced coagulopathy, but this needs to be studied at clinically relevant dosages [7-9]. Therefore, the purpose of this study was to evaluate the in vitro effects of mild hypothermia in the context of coagulopathy induced by haemodilution with crystalloid or hydroxyethyl starch solutions; and the effects of fibrinogen concentrate, alone or in combination with factor XIII concentrate using ROTEM, a well-known VHA. The primary hypothesis was that fibrinogen concentrate, with or without factor XIII, reduce dilutional coagulopathy and secondly that hypothermia attenuates their corrective effects.

## Material and Methods

### Volunteers

Ten healthy individuals (one woman and nine men, age 27–65 years) gave their written informed consent to participate in this study. None of the subjects received any medication in the preceding 7 days and none had a history of coagulopathy. The Regional Ethical Review Board in Lund, Sweden, approved the study (DNR 2008:484).

### Sampling

Blood was sampled into citrated plastic vacuum tubes (BD Vacutainer® Coagulation Tube, PET, 0.3 ml 0.109 M citrate). Venepuncture with minimal stasis was conducted, using a 21 Gauge needle, and the first tube was discarded. The samples were incubated at 33°C or 37°C for 30 minutes to ensure temperature equilibration and analysed within 4 hours of sampling.

#### Hypothermia and haemodilution

A digital thermometer submerged into a fluid-filled reference tube was used to check all blood sample temperatures. The ROTEM instruments were set at either 33°C or 37°C. The blood samples were diluted 2:1 volume by volume with either Ringer’s acetated solution (RAc; Fresenius Kabi, Bad Homburg, Germany(G)) or 6% hydroxyethyl starch in saline (HES; MW 130 kDa, substitution ratio 0.42, Venofundin®, B.Braun, Melsungen, G) that is 33% haemodilution.

### Fibrinogen and factor XIII

Human fibrinogen concentrate (Riastap®, CSL Behring Marburg, G) and factor XIII (FXIII) concentrate (Fibrogammin®; now registered as Cluvo®, CSL Behring) were dissolved according to the manufacturer’s instructions, giving concentrations of 20 mg/ml and 62.5 IU/ml respectively. 120 μl of fibrinogen concentrate or 120 μl of fibrinogen +15 μl of FXIII were added to the respective blood sample, to a total sample volume of 3000 μl. These dosages correspond to 4 g of fibrinogen and 1550 IU of FXIII to a 70-kg man or 55 mg fibrinogen and 22 IU FXIII per kg of body weight. The FXIII dosage followed recommendations from the manufacturer on how to treat haemorrhage in congenital FXIII-deficient patients (10–20 IU/kg of body weight). The 4 g fibrinogen dosage is in line with current guidelines on treatment of massive bleeding.

### ROTEM

Rotational thromboelastometry (ROTEM®; Pentapharm, Munich, Germany), a viscoelastic coagulation analysis instrument, was used according to the manufacturer’s instructions. Two ROTEM assays, EXTEM and FIBTEM, were used. Coagulation was stimulated with tissue factor in the EXTEM test and the following parameters were recorded: clotting time (CT), clot formation time (CFT), alpha-angle (AA) and maximal clot formation (MCF). The following EXTEM parameters measure the clot velocity: CT shows how long clot initiation takes while CFT and AA reflect the clot amplification and propagation. EXTEM-MCF measures the maximal clot strength and is dependent on platelet count and function, as well as fibrin formation and polymerisation. The FIBTEM test is identical to the EXTEM test except for that cytochalacin D, a platelet inhibitor is added to the test reagent. This results in FIBTEM-MCF representing clot strength dependent on fibrin formation and polymerization alone. The only FIBTEM parameter recorded was FIBTEM-MCF. The last parameter EX-FIBTEM-MCF is a surrogate measure of platelet activity, which is calculated as FIBTEM-MCF subtracted from EXTEM-MCF.

### Statistical analysis

For all statistical calculations the software package R, version 3.0.0, was used [10]. Repeated measures were analysed using univariate mixed models, using the package nlme ver. 3.1-109 [11]. Heterogeneous variances were evaluated with weighting. Post-hoc comparisons were made using the Multcomp package ver. 1.2-17 [12] using false discovery rate adjustment of p-values [13]. Data were analysed in two steps. The first step with haemodilution (control, RAc, HES) and two different temperatures (33°C, 37°C) sought to evaluate the potentially different effects of haemodilution with different solutions on normo- and hypothermic blood respectively. The second step used haemodilution with two different solutions (RAc, HES), two temperatures (33°C, 37°C) and the addition of coagulation factors (control, fibrinogen, fibrinogen + FXIII); this analysis evaluated the potential interaction between coagulation factors and the different solutions or different temperatures.

## Results

### Baseline values

In the undiluted samples, all EXTEM parameters and FIBTEM-MCF were within the normal range at 37°C [14].

#### Hypothermia and haemodilution

All clot velocity parameters were impaired by hypothermia of 33°C: in both undiluted and diluted blood CT and CFT were prolonged and AA was decreased (Figure 1). EXTEM-MCF decreased to a small extent during hypothermia, but only during concurrent haemodilution with either solution (Figure 2). Despite these hypothermic effects, the average values in undiluted samples were still mainly within the normal range at 33°C: a few samples’ EXTEM-CFT exceeded the reference range and EXTEM-AA and FIBTEM-MCF were below the reference range in a few of the samples.

**Figure 1:**
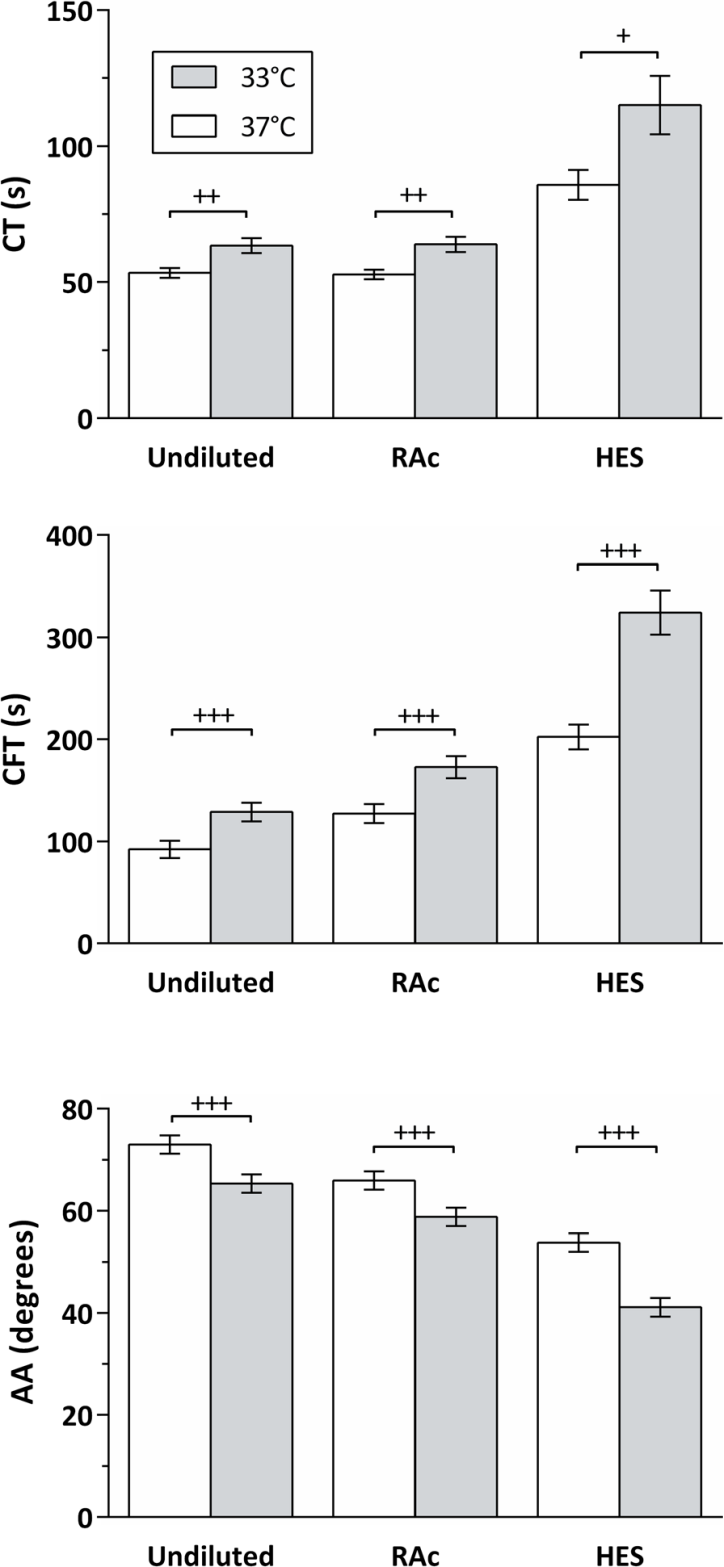
**Effects of hypothermia (33° vs. 37 C) and haemodilution on the ROTEM EXTEM parameters clotting time (CT), clot formation time CFT) and alpha angle (AA).** RAc: Ringer’s acetate, HES: hydroxyethyl starch. All differences between haemodilution groups (Undiluted vs. RAc, Undiluted vs. HES, and RAc vs. HES) within each temperature were significant (P < 0.001), except the differences of CT between Undiluted and RAc at both temperatures, which were not significant. Brackets show significant differences between temperatures. (+: *P* < 0.05, ++: *P* < 0.01, +++: *P* < 0.001). Data are presented as mean values, error bars are 95% simultaneous confidence intervals. N = 10.

**Figure 2:**
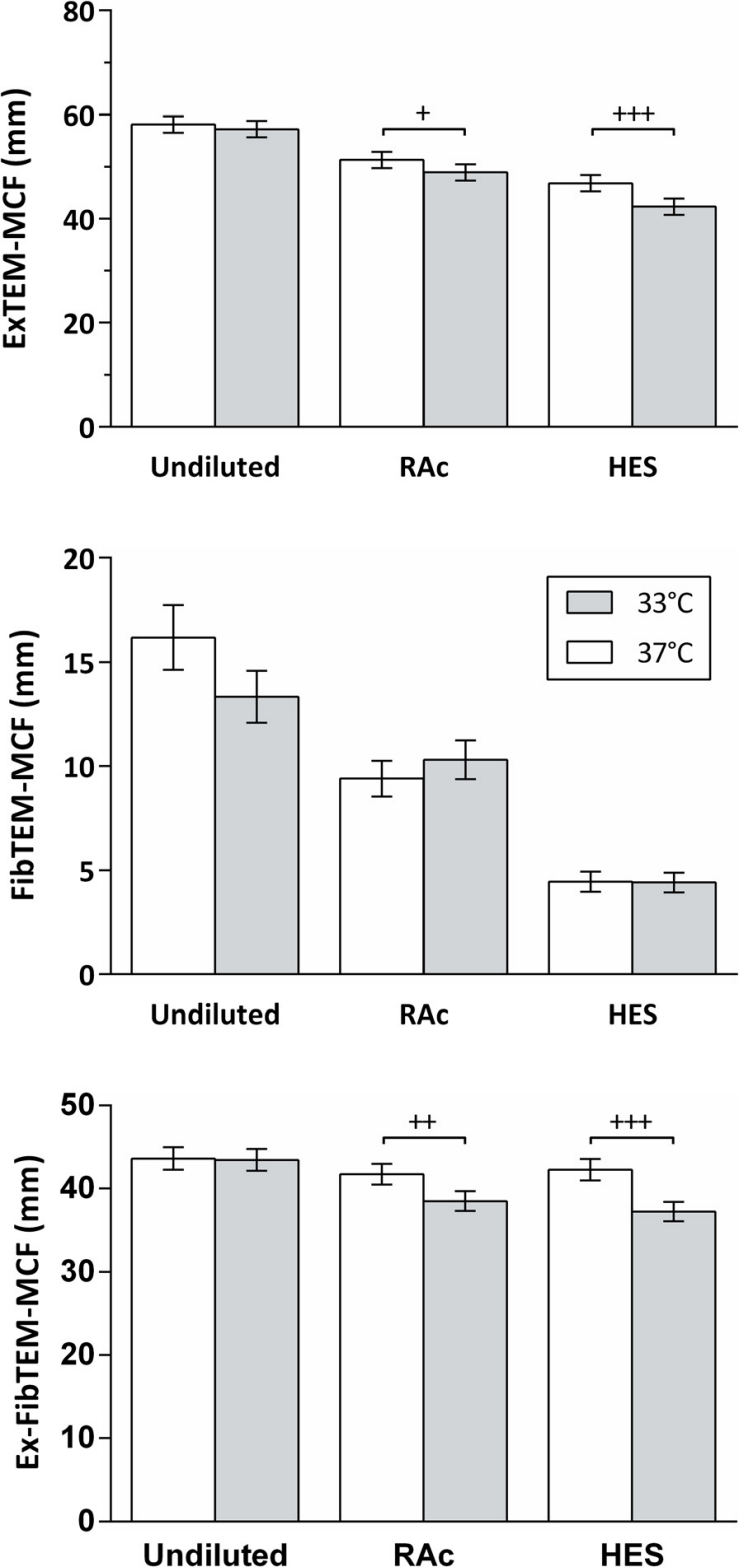
**Effects of hypothermia (33° vs. 37 C) and haemodilution on the ROTEM parameters.** EXTEM-MCF, FIBTEM-MCF and EX-FIBTEM-MCF; the latter calculated as FIBTEM-MCF subtracted from EXTEM-MCF. RAc: Ringer’s acetat, HES: hydroxyethyl starch. All differences of EXTEM-MCF and FIBTEM-MCF between haemodilution groups (Undiluted vs. RAc, Undiluted vs. HES, and RAc vs. HES) within each temperature were significant (*P* < 0.001), except the difference of FIBTEM-MCF between Undiluted and RAc at 33°C. Differences of EX-FIBTEM-MCF were only significant (*P* < 0.001) between Undiluted and haemodilution with either RAc or HES, at 33°C. Brackets show significant differences between temperatures. (+: *P* < 0.05, ++: *P* < 0.01, +++: *P* < 0.001). Data are presented as mean values, error bars are 95% simultaneous confidence intervals. N = 10.

At normothermia, haemodilution with HES significantly impaired all measured parameters, whereas RAc significantly impaired all parameters except CT. In addition, HES impaired ROTEM parameters significantly more than RAc (Figures 1 and 2). Moreover, the combination of hypothermia and HES haemodilution interacted to impair CFT and AA in relation to undiluted samples (CFT: *P* < 0.001 and AA: *P* < 0.05), as well as in relation to RAc haemodiluted samples (CFT: *P* < 0.001 and AA: *P* < 0.01).

### Addition of fibrinogen with or without factor XIII

Addition of fibrinogen concentrate to haemodiluted samples enhanced coagulation in general, except for CFT and AA in HES-haemodiluted samples. With FIBTEM-MCF and EXTEM-AA, there were significant interactions between fibrinogen concentrate and RAc haemodilution as compared to HES haemodilution, that is FIBTEM-MCF and AA were more effectively increased during RAc haemodilution than during HES haemodilution (*P* < 0.05 at both temperatures with FIBTEM-MCF and *P* < 0.05 at 37°C with AA) (Figures 3 and 4).

**Figure 3:**
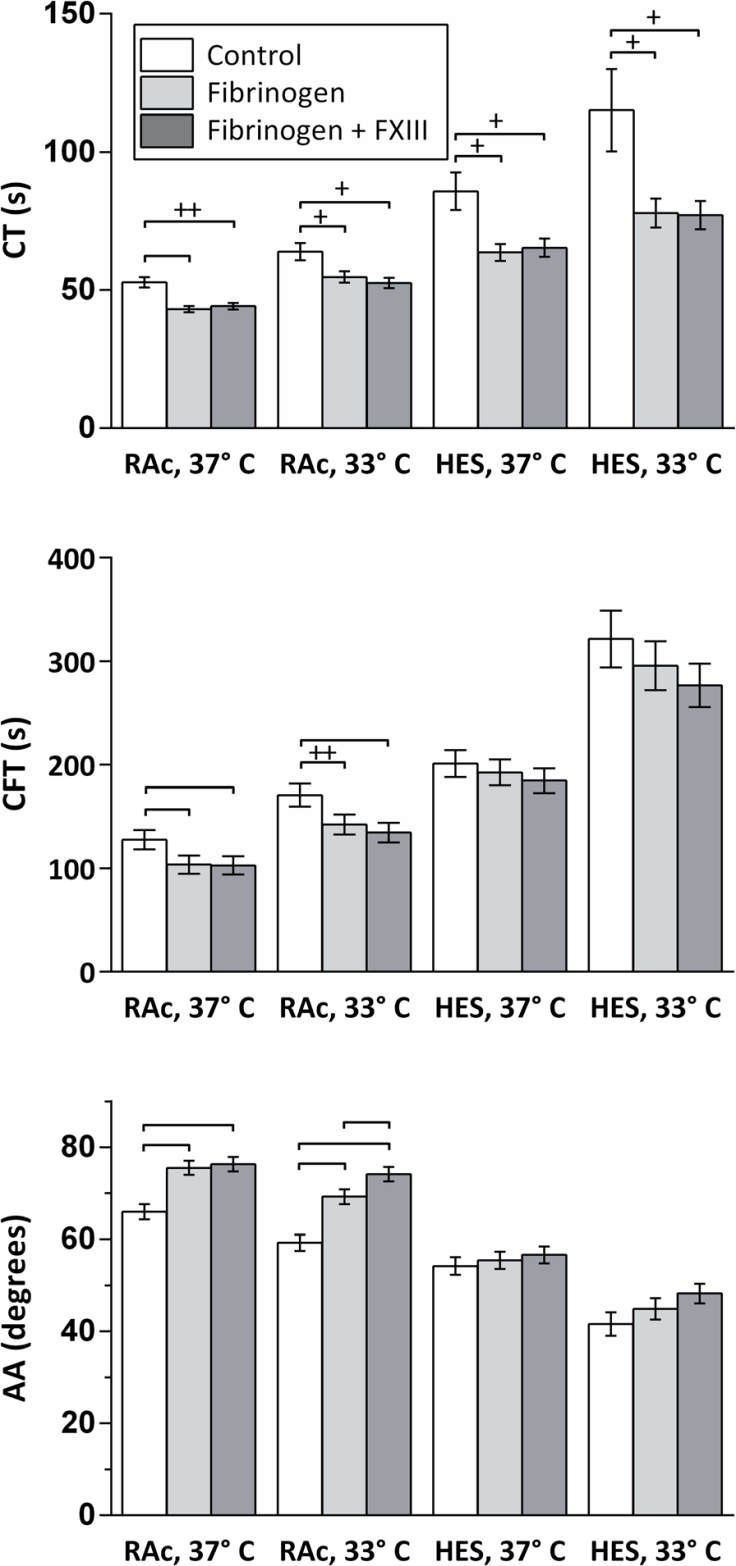
**Effects of coagulation factor concentrate (Fibrinogen or Fibrinogen with factor XIII (FXIII)) at two different temperatures (33° vs. 37 C) during haemodilution with Ringer’s acetate (RAc) or hydroxyethyl starch (HES).** ROTEM EXTEM parameters shown are clotting time (CT), clot formation time CFT) and alpha angle (AA). Statistically significant differences are marked with brackets. All significances are *P* < 0.001, except where elsewise stated; +: *P* < 0.05, ++: *P* < 0.01. Data are presented as mean values, error bars are 95% simultaneous confidence intervals. N = 10.

**Figure 4:**
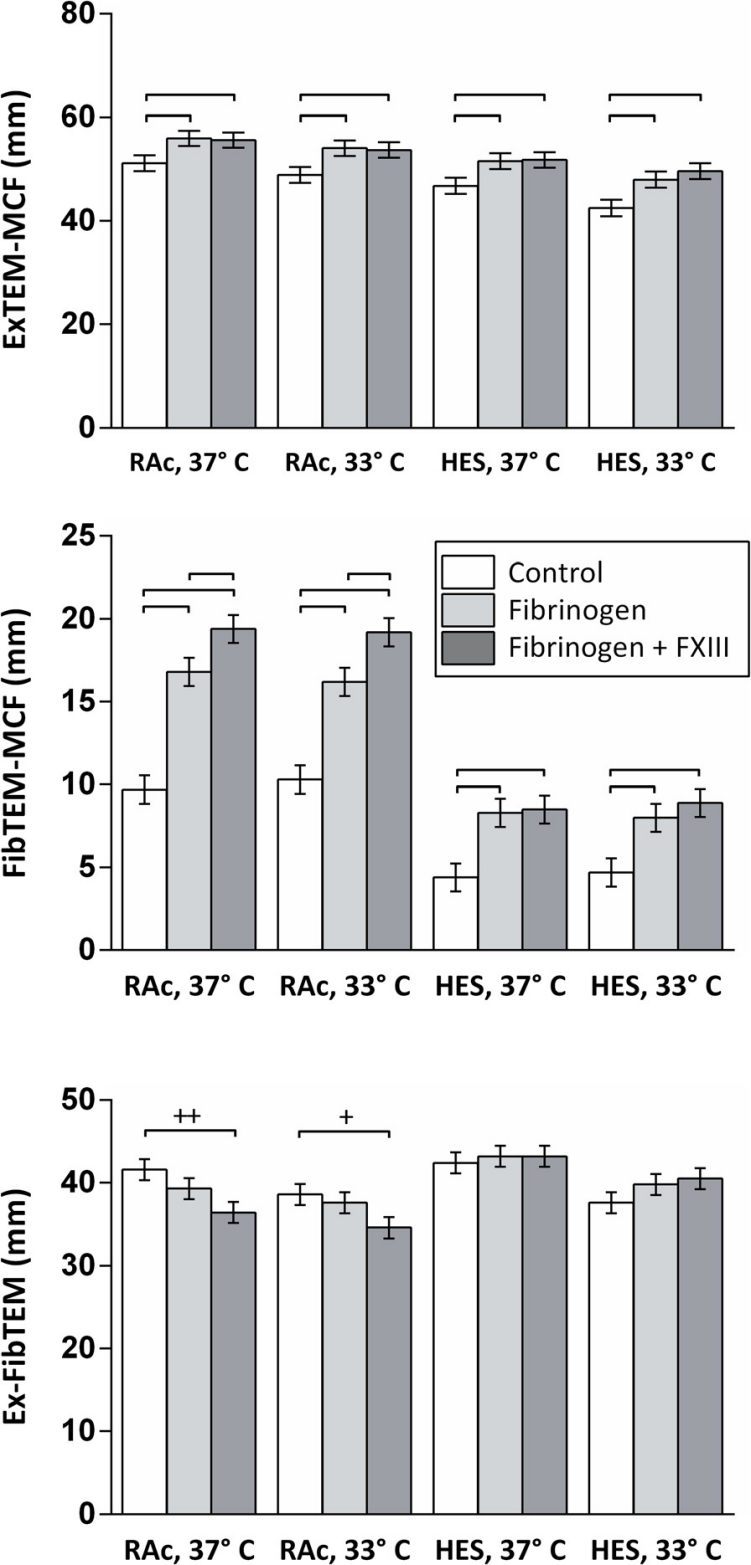
**Effects of coagulation factor concentrate (Fibrinogen or Fibrinogen with factor XIII (FXIII)) at two different temperatures (33° vs. 37 C) during haemodilution with Ringer’s acetate (RAc) or hydroxyethyl starch (HES).** ROTEM parameters shown are EXTEM-MCF, FIBTEM-MCF and EX-FIBTEM-MCF; the latter calculated as FIBTEM-MCF subtracted from EXTEM-MCF. Statistically significant differences are marked with brackets. All significances are *P* < 0.001, except where elsewise stated; +: *P* < 0.05, ++: *P* < 0.01. Data are presented as mean values, error bars are 95% simultaneous confidence intervals. N = 10.

Almost all the parameters were improved to the same degree by fibrinogen combined with FXIII, as by fibrinogen alone. However, FXIII had additional effects to the fibrinogen effects during RAc haemodilution; AA increased more at 33°C and FIBTEM-MCF increased more at both 33° and 37°C after addition of fibrinogen + FXIII as compared with fibrinogen alone (Figures 3 and 4). The better effect of fibrinogen combined with FXIII on FIBTEM-MCF is also demonstrated by significant synergies between RAc haemodilution and fibrinogen + FXIII as compared to control (*P* < 0.001 at both temperatures) as well as compared to fibrinogen alone (*P* < 0.05 at both temperatures).

### Platelet dependent clot strength (EX-FIBTEM-MCF)

The platelet dependent clot strength (EX-FIBTEM-MCF) was 72% and 76% of the EXTEM-MCF, at 37°C and 33°C respectively. Hypothermia at 33°C and haemodilution in combination decreased EX-FIBTEM-MCF, but hypothermia or haemodilution alone had no effect on EX-FIBTEM-MCF. HES and RAc affected EX-FIBTEM-MCF to the same extent (Figure 2). Addition of fibrinogen or fibrinogen + FXIII did not significantly change EX-FIBTEM-MCF with HES haemodilution, whereas EX-FIBTEM-MCF significantly *decreased* after adding fibrinogen + FXIII during RAc haemodilution (Figure 4).

## Discussion

### Principal findings

Our principal finding is that fibrinogen concentrate, with or without factor XIII concentrate, reduces dilutional coagulopathy irrespective of temperature (33° or 37°C) and that this effect was more pronounced with RAc than HES haemodilution. We also found that hypothermia affects coagulation more during haemodilution with HES than with RAc.

### Hypothermia and haemodilution

The effects of cooling undiluted blood to 33°C on ROTEM parameters were small. Although significant impairments of clot initiation (CT) and propagation (CFT, AA) were seen, measures of clot strength (EXTEM-MCF and FIBTEM-MCF) were unaffected. These results are in line with results from other in vitro studies with VHA’s, which demonstrate mild to moderate hypothermia to decrease the rate of clot formation but not the clot strength [15,16], even though more severe hypothermia (<28°C) may decrease clot strength [17]. An in vitro study on the relative effects of hypothermia on platelets and plasma coagulation indicated an impaired platelet adhesion as the primary cause of coagulopathy during mild hypothermia [2]. This may explain our previous results with free oscillation rheometry (FOR), which measured a decreased clot velocity as well as clot strength during hypothermia [9]. ROTEM is possibly better at detecting plasma coagulation than platelet function whereas FOR is more sensitive to platelet function.

The observations that haemodilution impaired parameters reflecting both the rate of clot formation and clot strength, and that HES attenuated these parameters more than RAc, is in line with previous research [18,19]. Numerous studies with VHA’s have shown a progressive decrease of initiation, amplification and propagation of coagulation as well as clot strength, where clot strength was more affected by synthetic colloids than crystalloids [9,20,21]. Synthetic colloids, such as gelatine and HES, interfere with fibrin network structure and consequently decrease clot strength more than explained by dilution of plasma proteins alone [22]. In contrast, some investigations have shown that haemodilution up to 50% with isotonic saline may induce a hypercoagulable state [23]. This has been suggested to be caused by decreased plasma antithrombin levels [24]. However, these studies used native non-activated blood, which may explain the difference from our results [25].

Hypothermia in combination with haemodilution mainly affected ROTEM parameters measuring clot velocity. Thus, HES haemodilution interacted with hypothermia to further impair CFT and AA. This is in line with our previous study with free oscillation rheometry (FOR) [9], but few other studies have addressed the combined effect of haemodilution and hypothermia. A study from 1994 measuring activated partial thromboplastin time found additional but not synergistic effects of hypothermia and haemodilution with a crystalloid [5]. Possible explanations for the observed synergy between hypothermia and HES, may be that both hypothermia [2,26] and HES [27,28] have direct effects on platelets, and that also plasma coagulation activity is decreased during both hypothermia [2] and haemodilution [29]. Platelet activation is very important for the thrombin burst associated with propagation of coagulation, which may explain why the ROTEM parameters CFT and AA were particularly affected by the combination of HES-haemodilution and hypothermia.

### Addition of fibrinogen with or without factor XIII

Our results imply that fibrinogen concentrate can be used to improve coagulation at 33°C. This was also suggested, albeit not statistically significantly, in our previous study with FOR [9]. The results from the present study also imply that a clot weakened by RAc is easier to improve than a clot weakened by HES, which corroborates results from previous studies [8,30,31]. Although fibrinogen also improved fibrinogen-dependent clot strength (FIBTEM-MCF) irrespective of fluid or temperature, this improvement was greater during RAc than HES haemodilution. The general view that hypothermia-induced coagulopathy is refractory to treatment with coagulation factor concentrates [32] is contradicted by our results, as well as by a previous study where fibrinogen concentrate increased whole blood coagulation at 32°C [33]. It is a principal finding of our study, that the clot stabilizing effect of fibrinogen concentrate on coagulopathy induced by RAc is as effective at 33°C as it is during normothermia. This implies that correction of hypothermia before substitution with fibrinogen concentrate is clinically not necessary.

Factor XIII improved fibrinogen’s effect on FIBTEM-MCF during RAc-haemodilution but not during HES haemodilution. In line with our study, several previous in vitro haemodilution studies on healthy volunteers have shown that although FXIII alone does not improve dilutional coagulopathy, it can enhance fibrinogen’s corrective effect [7-9,34]. These results contradict an in vitro study on blood from intensive care patients where supraphysiological doses of FXIII were used [35]. In this study, a high pre-existing concentration of fibrinogen in plasma may explain the corrective effect of FXIII alone. It is probably only worthwhile to administer factor XIII to correct a coagulopathy if normalizing plasma fibrinogen concentrations is cared for. Consequently, it may be of value to combine fibrinogen with factor XIII in order to improve clot stability during RAc-haemodilution.

### Platelet dependent clot strength (EX-FIBTEM-MCF)

The derived parameter EX-FIBTEM-MCF is considered to reflect platelet dependent clot strength. EX-FIBTEM-MCF decreased both after haemodilution and mild hypothermia. This is in line with studies using the cone and platelet analyser, where haemodilution [18] and mild hypothermia [2] showed reduced platelet aggregation. In contrast, some studies have found hypothermia to increase platelet aggregation, as measured with impedance aggregometry [36,37]. Aggregation is a process where fibrinogen stick platelets to each other through the platelet receptor glycoprotein IIb/IIIa, and fibrinogen concentrate has previously been shown to counteract glycoprotein IIb/IIIa–directed platelet inhibition [38]. We therefore expected the hypothermia induced reduction of platelet dependent clot strenght to be reversed by fibrinogen concentrate, but instead, EX-FIBTEM-MCF *decreased* significantly after the addition of fibrinogen + FXIII concentrates to RAc diluted blood. A recent study found platelet aggregation to be reduced or unaffected by in vitro supplementation with fibrinogen concentrate, depending on which platelet activator being used [39]. However, we believe that the reduction of EX-FIBTEM-MCF should not be interpreted as a decreased platelet function but that the addition of fibrinogen + FXIII increased FIBTEM-MCF values more than EXTEM-MCF, and hence the so called platelet dependent clot strength (EX-FIBTEM-MCF) decreased regardless of actual platelet function. There are principally two possible explanations of this shortcoming of ROTEM: 1) the ROTEM instrument may be more sensitive to changes at low levels of clot strength (MCF) than at normal levels; or 2) platelets are not fully inhibited by the FIBTEM assay. ROTEM uses forced oscillation and an arbitrary clot strength scale with a theoretical maximum of 100 mm, which makes a limited sensitivity at the upper scale possible. Platelet inhibition with cytochalacin D has been shown to be improved if combined with abciximab, a fibrinogen receptor inhibitor [40]. In conclusion, we and others question the reliability of EX-FIBTEM-MCF as a platelet function parameter [41] and suggest that other methods may be more suitable.

### Limitations

There are several methodological concerns when performing in vitro coagulation tests. For example, there is no activation of procoagulative or fibrinolytic responses secondary to tissue trauma [1]. Furthermore, shear strains applied with viscoelastic haemostatic assays such as ROTEM are substantially lower than those found in the human circulation, which may modify clot structure during measurements [42]. There are also concerns with in vitro haemodilution: acid–base regulation is not physiological and the decreased haematocrit may increase fibrin thread formation in the reaction chamber [43]. Therefore, we restricted dilution to 33%. Finally, there is much debate regarding the use of native whole blood versus citrated whole blood in VHAs. We used citrated blood, since it can be incubated and stored for up to four hours whereas native blood must be analysed within 4 minutes. However, incubation of citrated blood over 30 minutes has been shown to decrease thrombelastographic differences between native and citrate blood analyses [25].

## Conclusion

In conclusion, our results show that fibrinogen with or without FXIII corrected in vitro dilutional coagulopathy also during hypothermia and that the corrective effects were weaker during haemodilution with HES, as compared to RAc. Factor XIII enhanced the corrective effects of fibrinogen, but only during RAc haemodilution. Finally, HES haemodilution interacted with hypothermia to impair coagulation.

## Abbreviations

HES: Hydroxyethyl starch
RAc: Ringer’s acetate
ROTEM: Rotational thromboelastometry
CT: Clotting time
CFT: Clot formation time
AA: Alpha angle
MCF: Maximal clot formation
VHA: Viscoelastic haemostatic assays
FXIII: Factor XIII
FOR: Free oscillation rheometry
